# Effects of *Bacillus licheniformis* and Combination of Probiotics and Enzymes as Supplements on Growth Performance and Serum Parameters in Early-Weaned Grazing Yak Calves

**DOI:** 10.3390/ani13050785

**Published:** 2023-02-21

**Authors:** Jia Zhou, Kaiqiang Zhao, Lisheng Shao, Yuhong Bao, Dundup Gyantsen, Chenglong Ma, Bai Xue

**Affiliations:** 1 Key Laboratory of Low Carbon Culture and Safety Production in Cattle in Sichuan, Animal Nutrition Institute, Sichuan Agricultural University, Chengdu 611130, China; 2 Institute of Pratacultural, Tibet Academy of Agricultural and Animal Husbandry Sciences, Lhasa 850009, China; 3 Lhasa Poultry Breeding Research and Protection and Extension Center, Lhasa 850000, China

**Keywords:** early weaning, probiotics, enzyme preparations, yak calves, growth performance

## Abstract

**Simple Summary:**

This study was conducted to investigate the effects of dietary supplementation with Bacillus licheniformis and a combination of probiotics and enzymes on the growth and blood parameters of grazing yak calves. The body weight, body size, serum biochemical parameters, and growth hormone levels of grazing yaks were assessed. We found that supplementation with probiotics alone or with a combination of probiotics and enzymes significantly increased the average daily gain, compared to the controls, and the combination of probiotics and enzymes showed a better performance. Supplementation with the complex of probiotics and enzymes significantly increased the concentration of serum growth hormone, insulin-like growth factor-1, and epidermal growth factor, which may be the main reason for the higher daily weight gain. The findings of this study may help improve the growth efficiency of yak calves on the Qinghai–Tibetan Plateau.

**Abstract:**

Early weaning is an effective strategy to improve cow feed utilization and shorten postpartum intervals in cows; however, this may lead to poor performance of the weaned calves. This study was conducted to test the effects of supplementing milk replacer with *Bacillus licheniformis* and a complex of probiotics and enzyme preparations on body weight (BW), size, and serum biochemical parameters and hormones in early-weaned grazing yak calves. Thirty two-month-old male grazing yaks (38.89 ± 1.45 kg body weight) were fed milk replacer at 3% of their BW and were randomly assigned to three treatments (*n* = 10, each): T1 (supplementation with 0.15 g/kg *Bacillus licheniformis*), T2 (supplementation with a 2.4 g/kg combination of probiotics and enzymes), and a control (without supplementation). Compared to the controls, the average daily gain (ADG) from 0 to 60 d was significantly higher in calves administered the T1 and T2 treatments, and that from 30 to 60 d was significantly higher in calves administered the T2 treatment. The ADG from 0 to 60 d was significantly higher in the T2- than in the T1-treated yaks. The concentration of serum growth hormone, insulin growth factor-1, and epidermal growth factor was significantly higher in the T2-treated calves than in the controls. The concentration of serum cortisol was significantly lower in the T1 treatment than in the controls. We concluded that supplementation with probiotics alone or a combination of probiotics and enzymes can improve the ADG of early-weaned grazing yak calves. Supplementation with the combination of probiotics and enzymes had a stronger positive effect on growth and serum hormone levels, compared to the single-probiotic treatment with Bacillus licheniformis, providing a basis for the application of a combination of probiotics and enzymes.

## 1. Introduction

Yaks (*Bos grunniens*) occur on the Qinghai–Tibet Plateau at high altitudes and with long cold seasons and limited pasture resources. This species is a unique product of long-term natural selection, providing local herders with the most basic living materials and livelihood resources, such as meat, milk, shelter (hides and furs), and fuel (dung), and is an indispensable part of the ecology and economy of the Qinghai–Tibetan Plateau [1]. However, the low reproductive rate of yaks seriously restricts their production and utilization. The cold season on the Tibetan Plateau lasts for eight months (October to the following May), during which time the quantity and quality of pasture decrease below the nutritional requirements of lactating yaks [2]. The deficiency of feed intake results in a negative body energy balance and metabolic stress [3]. On the other hand, under traditional grazing management, plateau-grazing yak calves are weaned naturally or artificially under various conditions at an age of 18–24 months [4], rather than the weaning age of domestic beef cattle (<6 months). The slow recovery itself and the late weaning of yak calves, which result in a poor postnatal physical condition, severely affect the onset of the next estrous cycle in the cow. Most yaks exhibit a long postpartum anestrous period and calve twice every 3 years or once every 2 years [5]. Therefore, the early weaning of yak calves may help mitigate these adverse effects.

Early weaning has become more popular in recent years for various reasons, including the better use of limited feed resources and alleviating grazing pressure on pastures by reducing the nutritional needs of cows [6]. Weaning calves before the start of the breeding season improves the reproductive performance of cows [7,8] because the cows can regain their weight faster, thus accelerating the onset of postpartum estrus. The use of milk replacer in early weaning is common in livestock production [9,10]. The milk replacer has demonstrated positive benefits in animal experiments, such as improved immunity and relieved weaning stress response [11]. Increasing evidence suggests that enhanced milk replacer feeding is beneficial for improving gut microbial development and growth performance in early-weaned lambs [12,13].

Over the past few decades, probiotics have been widely used in livestock and poultry production for their ability to enhance animal disease resistance, improve feed utilization, and improve growth performance [14]. In ruminants, yeasts and bacteria, including *Lactobacillus*, *Bifidobacterium*, *Bacillus*, *Propionibacterium*, and *Enterococcus*, alone or in combination, are used as additives in diets [15,16]. Probiotics can decrease diarrhea, improve production and feed utilization efficiency, and strengthen the immunity system in young ruminants [17,18,19]. Moreover, supplementation with probiotics improves the rumen and intestinal epithelial cell growth, which enhances the gastrointestinal tract development and health status of calves [17,20,21]. Oral administration of *Bacillus licheniformis* can increase ruminal digestibility and total volatile fatty acid concentrations in Holstein cows [22] and growth performance in Holstein calves [23]. In vitro inoculation with *Bacillus licheniformis* also improves ruminal fermentation efficiency of forage of various qualities [24]. However, no information is currently available on the effect of *Bacillus licheniformis* on the growth performance of yak calves.

Compound enzyme preparations are produced from one or more preparations containing a single enzyme as the main entity, which is mixed or fermented with other single enzyme preparations to form one or more microbial products [25], including saccharylases, amylases, cellulases, proteases, phytases, hemicellulases, and pectinases. Depending on the differences in digestive characteristics and diet composition, specific enzyme preparations can be used for livestock [26]. Specific enzyme complex preparations can degrade multiple feed substrates (antinutrients or nutrients), and different types of enzymes can work synergistically to maximize the nutritional value of feed [27]. In buffalo calves, cellulase and xylanase are more effective with regard to average daily weight gain (ADG) and feed efficiency [28]. Further, the addition of exogenous fibrolytic enzymes to wheat straw has no effect on starter feed intake and increases nutrient digestibility and recumbency, but decreases the ADG of weaned Holstein dairy calves [29].

The effects of probiotics or compound enzyme preparations on the production performance and biochemical blood indexes of calves are not consistent [29,30,31,32,33]. The respective discrepancies may be due to differences in the amounts of added probiotics and exogenous enzymes, the strains of probiotics, diets, and animal management strategies. Therefore, this study was conducted to compare the effects of Bacillus licheniformis and a combination of probiotics and enzymes on the growth performance and serum parameters in yak calves, so as to provide a theoretical basis for the application of probiotics in grazing yak calves.

## 2. Materials and Methods

### 2.1. Animals and Treatment

This study was performed in accordance with the Chinese Animal Welfare Guidelines, and the experimental protocols were approved by the Animal Care and Ethics Committee of the Institute of Animal Husbandry and Veterinary Medicine, Tibet Academyof Agriculture and Animal Husbandry Science (No. #TAAAHS-2016–27).

The feeding trial was conducted at Damxung Co., (Lhasa, China; 30.5° N, 91.1° E) from July to October. The average altitude was 4200 m, the average annual temperature was 1.3 °C, and the average annual precipitation was 456.8 mm. Thirty two-month-old male yaks (38.89 ± 1.45 kg body weight (BW)) were fed milk replacer solution at 3% of their BW every day and were randomly assigned to three dietary supplementation treatments (*n* = 10, each), according to BW and age, as follows: T1, supplemented with 0.15 g/kg *Bacillus licheniformis* (2 × 10^10^ CFU/g); T2, supplemented with a 2.4 g/kg combination of probiotics and enzymes (containing 0.4 g/kg *Bacillus licheniformis*, 2 × 10^10^ CFU/g; 1.0 g/kg yeast, 1 × 10^10^ CFU/g; 1.0 g/kg mixture of xylanase, cellulase, and glucanase in a 1:1:1 ratio, xylanase, 20,000 U/g, cellulase, 1500 U/g, glucanase, 6000 U/g); and a control treatment. The milk replacer, probiotics, and enzyme preparations were provided by the Chinese Academy of Agricultural Sciences (Beijing, China). All yak calves were allowed to graze on an alpine meadow during daytime for the 60-day trial, and they were individually fed milk replacer before and after grazing (0800 and 2000 h, respectively). The forage of the alpine meadow was mainly composed of *Kobresia tibetica*, and the nutrient composition (dry matter basis) was analyzed in our previous study [34], i.e., 10.4% crude protein, 2.1% ether extract, 67.8% neutral detergent fiber, 34.2% acid detergent fiber, and 4.6% ash. The powdered milk replacer was weighed and mixed with warm water (approximately 40 °C) at a ratio of 1:7 (*w*/*v*) to obtain milk replacer solution, according to our previous study [35]. Based on preliminary assessments, the feeding amount of milk replacer was calculated so that all yak calves were able to feed without surplus [35]. The nutrient composition of the milk replacer is shown in Table 1.

### 2.2. Sample Collection and Analysis

The BW of each yak calf was recorded before morning feeding on d 0, 30, and 60 using a platform scale, and the ADG was calculated accordingly. The body size indexes of all yak calves were determined using a linen tape at the beginning (d 0) and end (d 60) of the experiment, as previously described [36].

Blood samples (approximately 10 mL) were collected from the jugular vein of the yak calves using a vacuum tube before morning feeding on d 0 and 60. The blood samples were centrifuged at 1100× *g* for 10 min to obtain serum, which was then aliquoted in 1.5 mL centrifuge tubes and stored at −20 °C.

The serum biochemical parameters, including blood urea nitrogen (BUN), globulin (GLB), blood glucose (GLU), and non-esterified fatty acids (NEFAs), were analyzed using an automatic biochemical analyzer 7020 (Hitachi, Tokyo, Japan). Metabolic hormones in the serum, including insulin-like growth factor-1 (IGF-1), epidermal growth factor (EGF), cortisol, insulin (INS), and growth hormone (GH), were determined using commercial ELISA kits (Jiahong Technology Co., Ltd., Beijing, China) according to the manufacturer’s instructions. Briefly, 50 μL of each five-fold diluted serum sample was added to each well of a 96-well ELISA plate. After 30 min of incubation at 37 °C, the plate was washed five times using PBS (Servicebio, Wuhan, China) to remove unbound proteins. Then, 50 μL of HRP-conjugated antibodies was added to allow them to bind with their corresponding antigens. The 3,3′,5,5′-tetramethylbenzidine working solution was added to each well, followed by stop solution. Absorbance was measured using a multi-plate reader (Varioskan LUX, Thermo Fisher Scientific, Waltham, MA, USA) at a wavelength of 450 nm.

### 2.3. Statistical Analysis

All experimental data of this study were statistically analyzed using a one-way analysis of variance followed by Duncan’s post hoc test with SPSS 26.0 software (SPSS Inc., Chicago, IL, USA). Each yak calf was considered an experimental unit. Data are expressed as means ± standard error. *p* < 0.05 was considered statistically significant.

## 3. Results

### 3.1. Body Weight

The three treatments did not differ significantly in terms of BW on d 0, 30, and 60 (Table 2). The ADG was higher (*p* < 0.05) in the calves under T2 treatment than those under the control treatment, from d 0 to 30, d 30 to 60, and d 0 to 60, and higher (*p* < 0.05) than that of those calves under the T1 treatment from d 0 to 60, indicating that the supplementation of *Bacillus licheniformis* and the combination of probiotics and enzymes could improve the growth performance of early-weaned grazing yak calves. The ADG of calves under T1 treatment was higher (*p* < 0.05) than that of those under the control treatment from d 0 to 60.

### 3.2. Body Size

The body size parameters did not differ significantly among the three treatments on d 0 and 60 (Table 3), indicating that the supplementation of *Bacillus licheniformis* and the combination of probiotics and enzymes did not affect the body size of yak calves within 60 d.

### 3.3. Serum Biochemical Parameters

The concentrations of serum GLB, BUN, GLU, and NEFAs did not differ significantly among the three treatments on d 0 and 60 (Table 4).

### 3.4. Serum Hormone

As shown in Table 5, the concentrations of serum IGF-1 on d 60 were higher in T2-treated calves than in the T1- and control-treated calves (*p* < 0.05, each). The concentrations of serum EGF and GH on d 60 were higher in the T2-treated calves than in the controls (*p* < 0.05). The concentration of serum COR on d 60 was higher in the control calves than those under the T1 treatment (*p* < 0.05).

## 4. Discussion

Early weaning may have various benefits for cows; however, early weaned calves generally perform poorly compared to naturally weaned calves [37]. Early weaned calves without breastfeeding grew at a lower rate and subsequently took longer to reach their target weight than breastfed calves [38]. To improve the growth performance of early-weaned calves, several improvements were made to the composition of milk replacer or additional feeds were added [39,40,41]. Moreover, the addition of probiotics to the diets of calves significantly improved the ADG [29,30,33]. Dietary supplementation with compound enzyme preparations also improved growth performance in weaned piglets [42,43] and growing-finishing pigs [44]. However, previous studies also reported that supplementation with probiotics, yeast cultures or enzymes had no effect on the growth performance of calves [31,32,45]. In the current study, the addition of *Bacillus licheniformis* alone or a complex of probiotics and compound enzyme preparations to the milk replacer significantly improved the performance of grazing yaks and calves compared with milk replacer alone. Further, the addition of probiotics is beneficial for the regulation of the intestinal microbiota community structure, improving intestinal health and fecal consistency, and reducing diarrhea prevalence [19,31,46,47,48]. The supplementation of fibrolytic enzyme to the diet of crossbred calves improved their nutrient digestibility with a positive effect on daily gain [49]. Calves typically exhibit high metabolism and fast growth; however, their growth performance is susceptible to environmental stress and nutrient absorption and digestive problems, especially in the period after weaning [50]. Under natural grazing conditions on the Qinghai–Tibet Plateau, due to the long-term lack of pasture and harsh environmental conditions, the normal growth of yak calves is severely restricted [48]. In the present study, none of the study animals died, which may be attributed to the supplementation with milk replacer. Therefore, the addition of probiotics and compound enzyme preparations was beneficial for the growth of grazing yak calves.

In most cases, calf weight is positively correlated with body length, and body length can be used to predict calf live weight [51,52]. Supplementation with *Bacillus subtilis* results in an increased body length and BW in Barki lambs at the third and fourth week, as observed in a four-week continuous feeding trial [53]. In the present study, neither body size nor BW differed among the treatments, which may be due to insufficient trial duration and individual differences in animals. Therefore, more time may be required to elucidate whether the probiotic and compound enzyme preparations affected the calves’ body size.

To a certain extent, blood biochemical parameters reflect the metabolism and the acid–base balance of the animal body, and they vary within a certain range [54,55]. The results of the current study revealed that supplementation with *Bacillus licheniformis* and the complex of probiotics and enzyme preparations had no effect on the blood biochemical parameters of grazing yak calves, which is consistent with previously reported results in crossbred and Holstein calves [56,57]. The blood biochemical values of calves vary with the growing stage and are strongly influenced by weaning [58,59], and these possible factors may be stronger than the influence of diet on blood biochemical indicators.

Insulin-like growth factors (IGFs) are small polypeptide hormones mainly synthesized and secreted from the liver, and they are structural homologs of insulin, with similar activities. These consist in binding to specific carrier proteins in the blood to form a composite factor that stimulates systemic body growth and has growth-promoting effects on almost every cell in the body [60,61]. As mediators of GH action, the synthesis of IGFs is also affected by the blood level of GH [62]. EGF is a member of the growth factor family, a single polypeptide of 53 amino acid residues that is involved in regulating cell proliferation [63]. We found that the addition of probiotics and a combination of probiotics and enzymes significantly increased the concentration of serum IGF-1, EGF, and GH, whereas supplementation with *Bacillus licheniformis* alone did not achieve this effect. These results are consistent with the ADG results. GH and IGF-1 are important controllers in regulating amino acid metabolism in calves, where GH promotes the entry of amino acids in muscle tissue into cells and increases protein synthesis, and IGF-1 increases protein deposition by promoting protein synthesis [63,64]. Cortisol is commonly used as a marker of stress responses (such as weanling stress) in animals, and it occurs at high serum levels for a period of time after calves are weaned [65]. In line with our results, oral supplementation with probiotics markedly decreases the concentrations of serum cortisol in neonatal and weaned calves [66,67]. Interestingly, we found that the concentrations of serum cortisol were lower in the T1 than in the T2 group, which was, however, not statistically significant. This suggested that the addition of *Bacillus licheniformis* alone may better alleviate weaning stress in grazing yak calves. However, the respective mechanisms remain to be resolved in more detail.

A limitation of this study is that the T2 group did not strictly control a single variable compared to the T1 group, and the factors (yeast or xylanase, cellulase and glucanase) that contributed to the difference were unclear. This was due to the initial intention of this study to improve the milk replacer by adding probiotics or compound enzyme preparations, and ultimately promote the growth performance of yak calves on the Qinghai–Tibet Plateau. Further, we were unable to collect data on diarrhea and determine nutrient digestibility in grazing calves, which would have further improved our understanding of the weight gain of yaks under the various treatments.

## 5. Conclusions

Our results suggest that supplementation with *Bacillus licheniformis* alone or with a complex of probiotics (*Bacillus licheniformis* and yeast) and compound enzyme preparations (xylanase, cellulase, and glucanase) can improve the ADG of grazing yak calves, and the complex had a better effect on the ADG. The addition of the complexes of probiotics and complex enzyme preparations also increased the concentrations of serum GH, IGF-1, and EGF, which may have led to a higher ADG. Thus, the addition of a combination of probiotics and enzymes to milk replacer may serve as an effective strategy to improve the production of yak calves.

## Figures and Tables

**Table 1:** Ingredients and nutrient compositions of milk replacer powder (feed basis, %).

Items	Nutrient Composition ^1^
Dry matter, %	96.22
GE, MJ/kg	19.66
Crude protein, %	26.11
Ether extract, %	25.66
Ash, %	7.23
Calcium, %	1.17
Total phosphorus, %	0.65

^1^ All nutrients were measured values.

**Table 2:** Effect of *Bacillus licheniformis* and the combination of probiotics and enzymes on body weight of yak calves.

Items	Treatments ^1^			SEM	*p*-Value
	Control	T1	T2		
Body weight, kg					
0 d	40.64	40.13	40.07	1.00	0.956
30 d	44.27	44.63	45.7	1.29	0.775
60 d	48.09	49.42	51.47	1.48	0.367
Average daily gain, g					
0–30 d	121.21 ^b^	150.00 ^ab^	187.78 ^a^	11.32	0.009
30–60 d	127.27 ^b^	159.72 ^ab^	192.22 ^a^	9.18	0.006
0–60 d	124.24 ^c^	154.86 ^b^	190.00 ^a^	8.88	<0.001

^1^ Control, yak calves supplemented with milk replacer solution of 3% BW; T1, yak calves supplemented with milk replacer solution according to BW plus 0.15 g/kg *Bacillus licheniformis*; T2, yak calves supplemented with milk replacer solution according to BW plus 2.4 g/kg combination of probiotics and enzymes; data with different lower-case superscript letters are significantly different (*p* < 0.05); *n* = 10; SEM, standard error of the mean.

**Table 3:** Effect of *Bacillus licheniformis* and the combination of probiotics and enzymes on body size of yak calves.

Items	Treatments ^1^			SEM	*p*-Value
	Control	T1	T2		
Body length, cm					
d 0	52.23	51.93	52.07	0.72	0.915
d 60	59.69	61.63	62.16	0.68	0.126
Hip height, cm					
d 0	60.59	59.84	60.77	0.82	0.788
d 60	64.54	63.72	64.15	0.35	0.815
Heart girth, cm					
d 0	69.45	69.36	68.99	0.26	0.925
d 60	78.51	78.48	78.42	0.28	0.932

^1^ Control, yak calves supplemented with milk replacer solution of 3% BW; T1, yak calves supplemented with milk replacer solution according to BW plus 0.15 g/kg *Bacillus licheniformis*; T2, yak calves supplemented with milk replacer solution according to BW plus 2.4 g/kg combination of probiotics and enzymes; *n* = 10; SEM, standard error of the mean.

**Table 4:** Effect of *Bacillus licheniformis* and the combination of probiotics and enzymes on serum biochemical parameters of yak calves.

Items	Treatments ^1^			SEM	*p*-Value
	Control	T1	T2		
GLB, mg/mL					
d 0	39.67	38.12	42.21	1.98	0.856
d 60	40.99	41.49	45.18	3.46	0.455
BUN, mmol/L					
d 0	24.29	26.15	24.89	1.21	0.658
d 60	27.12	24.86	25.24	1.00	0.627
GLU, mmol/L					
d 0	6.67	7.15	7.59	0.24	0.521
d 60	7.57	6.80	7.26	0.27	0.524
NEFAs, μmol/L					
d 0	566.74	591.26	543.36	16.59	0.642
d 60	667.63	583.54	599.50	25.71	0.377

^1^ Control, yak calves supplemented with milk replacer solution according to BW; T1, yak calves supplemented with milk replacer solution according to BW plus 0.15 g/kg *Bacillus licheniformis*; T2, yak calves supplemented with milk replacer solution according to BW plus 2.4 g/kg combination of probiotics and enzymes; GLB, globulin; BUN, blood urea nitrogen; GLU, blood glucose; NEFAs, non-esterified fatty acids; *n* = 10; SEM, standard error of the mean.

**Table 5:** Effect of *Bacillus licheniformis* and the combination of probiotics and enzymes on serum hormone of yak calves.

Items	Treatments ^1^			SEM	*p*-Value
	Control	T1	T2		
IGF-1, ng/mL					
d 0	273.52	295.12	287.36	18.95	0.195
d 60	156.25 ^b^	208.69 ^b^	319.38 ^a^	29.55	0.001
EGF, pg/mL					
d 0	846.26	926.21	912.62	30.15	0.389
d 60	429.82 ^b^	608.88 ^ab^	729.04 ^a^	40.71	0.007
GH, ng/mL					
d 0	17.59	14.25	15.77	1.68	0.625
d 60	7.94 ^b^	11.77 ^ab^	14.12 ^a^	0.89	0.012
INS, mIU/L					
d 0	28.96	27.21	26.85	1.26	0.851
d 60	26.32	22.66	29.76	3.85	0.764
COR, ng/mL					
d 0	107.26	101.68	116.26	2.65	0.856
d 60	105.42 ^a^	80.79 ^b^	93.49 ^ab^	4.29	0.049

^1^ Control, yak calves supplemented with milk replacer solution according to BW; T1, yak calves supplemented with milk replacer solution according to BW plus 0.15 g/kg *Bacillus licheniformis*; T2, yak calves supplemented with milk replacer solution according to BW plus 2.4 g/kg combination of probiotics and enzymes; IGF-1, insulin-like growth factors-1; EGF, epidermal growth factor; GH, growth hormone; INS, insulin; data with different lower-case superscript letters are significantly different (*p* < 0.05); *n* = 10; SEM, standard error of the mean.

## Data Availability

The data presented in this study are available on request from the authors.
